# Molecular epidemiology and population structure of *Providencia stuartii* obtained from humans and other sources

**DOI:** 10.1128/spectrum.02032-25

**Published:** 2025-12-31

**Authors:** Nayeli Estefania Sánchez-Casiano, Nadia Rodríguez-Medina, Edgar Aguilar-Vera, Alejandro Aguilar-Vera, Luisa María Sánchez-Zamorano, Enrique Delgado-Suárez, Erendira Cervantes-Caballero, Rayo Morfi-Otero, Eduardo Rodríguez-Noriega, Esteban Gonzalez-Diaz, Carlos Antonio Couoh-May, Luis Esaú López-Jacome, María del Consuelo Velázquez-Acosta, Cecilia Padilla-Ibarra, Elena Victoria Choy-Chang, Alejandro Alvarado-Delgado, Elvira Garza-González, Jesus Rojas, Esperanza Martínez-Romero, Ulises Garza-Ramos

**Affiliations:** 1 Instituto Nacional de Salud Pública, Centro de Investigación Sobre Enfermedades Infecciosas, Laboratorio de Resistencia bacteriana 37764, Cuernavaca, Morelos, México; 2 Programa de Doctorado en Ciencias Bioquímicas, Universidad Nacional Autónoma de México 7180, Mexico City, México; 3 Universidad Nacional Autónoma de México, Centro de Ciencias Genómicas 7180, Cuernavaca, Morelos, México; 4 Instituto Nacional de Salud Pública, Centro de Investigación en Salud Poblacional 103615, Cuernavaca, Morelos, México; 5 Universidad Nacional Autónoma de México. Facultad de Medicina Veterinaria y Zootecnia. 7180, Ciudad de México, México; 6 Universidad de Guadalajara, Instituto de Patología Infecciosa y Experimental, Hospital Civil de Guadalajara Fray Antonio Alcalde 27802, Guadalajara, México; 7 Hospital General Dr. Agustin O’Horán, Yucatán, México; 8 Instituto Nacional de Rehabilitación Luis Guillermo Ibarra 61663 , Ciudad de México, México; 9 Instituto Nacional de Cancerología 42597, Ciudad de México, México; 10 Hospital General del Estado Dr. Ernesto Ramos Bours 385683 , Sonora, México; 11 Hospital General de Zona No.1 IMSS Nueva Frontera, Tapachula, Chiapas, México; 12 Universidad Autónoma de Nuevo León, Departamento de Bioquímica y Medicina Molecular 27771, Monterrey, Nuevo León, México; 13 Instituto Nacional de Salud Pública, Centro de Información para Decisiones en Salud Pública 103619, Cuernavaca, Morelos, México; Sacramento County Public Health Laboratory, Sacramento, California, USA

**Keywords:** Gram-negative bacteria, phylogenetic analysis, molecular typing, healthcare-associated infections, intrinsic resistance, acquired resistance to carbapenem

## Abstract

*Providencia stuartii* is an emerging opportunistic pathogen in humans and belongs to a genus that has shown great genomic diversity. *P. stuartii* has shown relevant traits in antimicrobial resistance because of its intrinsic resistance to multiple antibiotics and its ability to acquire resistance genes that limit therapeutic options. The present study describes the molecular epidemiology of *P. stuartii* based on the multilocus sequence typing (MLST) scheme developed for this purpose. This non-PCR-based scheme uses seven complete housekeeping genes. In total, 518 genomes obtained from public databases and Mexican isolates were evaluated. These genomes were from different sources, such as human infections, followed by insects, wastewater, animals, and unknown origins. The molecular epidemiology showed 104 distinct sequence types (STs). We identified global clones that are multidrug-resistant and are widely geographically distributed; for instance, the ST72 (belonging to main clonal complex [CC1]), the major sequence type in *P. stuartii*, ST12 was present in human and non-human niches, and the ST19 and ST90 (present in Mexico) are associated with NDM-1 and the IncA/C2 plasmid replicon. This study provides a tool for a more accurate analysis of the molecular epidemiology of *P. stuartii* obtained from different sources and revises its genetic diversity and global distribution. In addition, this work developed a public web platform (http://mlstps.insp.mx) that implements average nucleotide identity tool for correct species classification followed by the ST assignation. This MLST scheme might be useful for future studies that focus on the evolution of this pathogen and monitoring the global problem clones.

**IMPORTANCE:**

*Providencia stuartii* is of particular concern due to its natural resistance to antibiotics, such as colistin and tigecycline, as well as its increasing resistance to carbapenems. Consequently, *P. stuartii* is an emerging pathogen that has been reported as a cause of human infections and is also distributed in non-human niches such as insects, wastewater, and animals. To understand how bacteria spread, researchers study their “genetic fingerprint” using methods like multilocus sequence typing (MLST), which for decades has been a useful approach for studying the molecular epidemiology of pathogens. Therefore, in this study, we studied the genetic diversity of *P. stuartii* implementing an MLST scheme designed for this purpose. We identified multidrug-resistant clones that are distributed worldwide. This system could be useful for future studies focusing on the evolution of this pathogen and monitoring high-risk clones. Tracking these strains is important for protecting public health and preventing outbreaks.

## INTRODUCTION

*Providencia* is a genus of Gram-negative bacteria belonging to the Enterobacterales order, the *Morganellaceae* family. It forms part of the gut microbiota of humans and some animals (1). Recently, advances in genomic studies have revealed remarkable diversity within the genus, creating major challenges for its taxonomic classification (2, 3). These difficulties are reflected in the misidentification of strains using conventional methods, such as MALDI-TOF, which were subsequently recognized as new species (4–6) A clear example is *P. thailandensis*, which years later was confirmed through genomic analyzes to correspond to *P. stuartii* (4). In addition, a comprehensive genomic analysis by Dong et al. (4) integrated average nucleotide identity (ANI) with phylogeny to reassess the taxonomy of the genus *Providencia*. This study reclassified 545 genomes into 20 species, including 13 previously described taxa and seven new unnamed taxa (Taxa 1–7) that could not be assigned to existing species. This analysis revealed extensive inconsistencies in species labeling, biochemical misidentification, and overall genomic diversity within the genus, underscoring the need to reevaluate the current classification system. Subsequently, the same group described additional novel species that further expanded the genus (5–7).

Currently, the genus includes 16 main species publicly validated and corrected under the International Code of Nomenclature of Prokaryotes (ICPN) according to the list of prokaryotic names (8) consulted in August 2025 (Table S1). Among these, *P. stuartii* is one of the most clinically relevant species, being a major causative agent of healthcare-associated infections and rarely with community-acquired cases. It predominantly affects immunocompromised patients and can cause pneumonia, soft tissue infections, wound infections, bloodstream infections, endocarditis, meningitis, and peritonitis. *P. stuartii* is notably linked to urinary tract infections, where it contributes significantly to morbidity and mortality often requiring prolonged antimicrobial treatment (9, 10). Beyond the clinical setting, *Providencia* species have been isolated from diverse ecological niches, facilitating their interaction with humans, animals, and the environment, and promoting their dissemination. Of particular concern is their intrinsic resistance to colistin and tigecycline combined with their ability to acquire plasmid-borne carbapenemase genes (11) representing a growing threat to public health.

Genomic analyses provide a valuable reference for precise taxonomic resolution within *Providencia*. Multilocus sequence typing (MLST) is the gold standard molecular tool for establishing the epidemiology of isolates across different geographic regions enabling the identification of potential relationships and emergence of pandemic or regionally dominant clones within these species (12). In the present study, a regional epidemiological surveillance was carried out to determine the regional and global molecular epidemiology of *P. stuartii*, providing the basis for a new MLST scheme and public database for this bacterial species (http://mlstps.insp.mx).

## MATERIALS AND METHODS

### Regional *Providencia* spp. epidemiological surveillance

This work was part of a surveillance study of *Providencia* spp. that was conducted in two periods; the first period was from 2012 to 2018 and the second from 2019 to 2024. Fig. S1 details the workflow carried out in both periods. The surveillance study was in collaboration with several institutions and hospitals in Mexico that provided the strains (Table S2). A total of 160 clinical isolates were obtained and identified by automatized systems using VITEK 2 (BioMérieux, Marcy l'Etoile, France) or MALDI-TOF MS (Bruker Daltonics, Bremen, Germany) as *P. rettgerii* (90 isolates) and *P. stuartii* (70 isolates). The clinical characteristics of *P. stuartii* are described in Table S3.

### Characterization of *P. stuartii* clinical isolates

The 70 clinical isolates of *P. stuartii* were evaluated using the Carba NP test following the recommendations of the Clinical and Laboratory Standards Institute (CLSI; 2023) (13). Carbapenemase-encoding genes (NDM, VIM, IMP, and OXA-like families) were screened by polymerase chain reaction (PCR) (Table S4).

All isolates were genotyped by pulsed-field gel electrophoresis (PFGE) (14). Genomic DNA was digested with *SfiI,* and clonal relatedness was assessed according to Tenover’s criteria. Clusters were defined as DNA banding patterns sharing >80% similarity. Dendrograms were generated using Dice and UPGMA coefficients implemented in GelCompare Software II, v.6.6.11 (Applied Maths, Inc.; Sint-Martens-Latem, Bélgica).

Plasmid DNA from all strains was extracted using alkaline lysis protocol described by Kiesser (15), and plasmid profiles were visualized by agarose gel electrophoresis. *Escherichia coli* NCTC50192, which carries plasmids of known size, served as the molecular weight reference. Conjugation assays were performed following the method described by Miller (16) to assess the transferability of plasmid-borne NDM gene. Sodium azide-resistant *E. coli* J53 was used as the recipient strain. Transconjugants were selected on LB agar containing 100 μg/mL sodium azide and 2 μg/mL imipenem. PCR amplification for NDM and plasmid extraction in transconjugants was performed to confirm plasmid transfer.

### MLST scheme development

#### Whole-genome sequencing, assembly, and annotation

Isolates selected for whole genome sequencing (WGS) were chosen based on PFGE fingerprint patterns, sample origin, year of collection, isolation site, resistance genes, and plasmid content (Fig. S2). One and 26 *P*. *stuartii* isolates were selected from the first and second surveillance periods, respectively (Fig. S1). WGS of these 27 isolates was performed using the Illumina NextSeq platform. Read quality was assessed using FastQC v0.12.1, and adapter trimming and quality filtering were performed with TrimGalore v0.6.5 (15). Genomes were assembled using Unicycler v3 in bold mode. The species assignation was confirmed by ANI (17) using pyani v0.2.x, and by maximum likelihood (ML) phylogeny reconstruction using core-genome single-nucleotide polymorphisms (SNPs) identified with Snippy v2 (18). Genomes that showed ANI values between 84% and 85% and formed a different clade were excluded from further analysis (5 genomes) (Fig. S1).

The *P. stuartii* genomes were deposited in GenBank under the BioProject “PRJNA1272119”. The genome of the strain 15,300 was included in the development of the MLST scheme, and the remaining 21 genomes served for MLST validation (Fig. S1).

#### Selection and identification of *P. stuartii* genomes from public databases for MLST development

In January 2024, a total of 638 *Providencia* spp. genomes were obtained from RefSeq database, which were selected and subjected to quality control using CheckM v2. The taxonomy classification was defined jointly by ANI analysis and ML phylogeny of the core genome SNPs using Snippy v2. We used snippy-multi and snippy-core options to generate the core genome SNP alignment. ML phylogenetic reconstruction was carried out using IQ-TREE under the GTR + I + G substitution model with 1000 bootstrap replicates and visualized in iTOL v6 web platform (Interactive Tree of Life) (19). A total of 95 genomes that displayed ANI values >99% relative to *P. stuartii* reference genome and the strain 15300 were used to develop the MLST scheme (Fig. S1b).

In addition, a pan-genome analysis was performed for genomes labeled as *P. stuartii*. Gene annotation was performed using Prokka v.1.13.4 (20) with the --prodigaltf option, and the resulting GFF files were used as input for Panaroo v.1.2.8 (21), which was run with –clean-mode strict and –remove-invalid-genes options. The core alignment generated was used to construct a ML phylogeny with IQ-TREE (GTR + I + G model). The phylogeny and the gene content data were then visualized with Phandango v. 1.3.1 (22).

#### Development of MLST scheme for *P. stuartii*

Core genes were extracted from the 96 genomes identified as *P. stuartii* from the previously described pan-genome analysis. A total of 2,814 core genes were identified, and after filtering out gene duplications, hypothetical or putative proteins, and genes smaller than 600 bp, a total of 987 core genes were obtained. These genes were analyzed using DnaSP v6.12.03 (23) to determine genetic diversity and identify housekeeping genes evolving under negative selection. Seven single-copy genes, *rpoB, leuS, pflB, dnaA, ftsA, sucC,* and *tsf,* were selected to be considered in the *P. stuartii* MLST scheme. The number of alleles and nucleotide diversity are detailed in Table 1.

**TABLE 1:** Characteristics of genes, alleles, and nucleotide diversity for the *P. stuartii* MLST scheme

Gene	Function	Size (bp)^*a*^	No. of alleles	Nucleotide diversity
*rpoB*	RNA polymerase subunit β	4,029	58	0.00282
*leuS*	leucyl-tRNA synthetase	2,583	48	0.00529
*pflB*	Formate acetyltransferase 1	2,283	49	0.00412
*dnaA*	Chromosomal replication initiator protein DnaA	1,392	20	0.00336
*ftsA*	Cell division protein FtsA	1,257	19	0.00362
*sucC*	Succinyl-CoA synthetase subunit β	1,167	13	0.00283
*tsf*	Elongation factor Ts	852	16	0.00386

^*a*^ The gene size corresponds to the complete gene in the *P. stuartii* bacteria species.

#### Sequence type determination and validation of MLST scheme

The sequence types (STs) were determined for the 96 *P*. *stuartii* genomes using the MLST scheme developed in this study. The human strain *P. stuartii* 15300 obtained in the first surveillance period (2018) was assigned as the ST1. Subsequent STs were assigned chronologically based on isolation dates available in the BioSample metadata for each genome in RefSeq genome (Data set S1).

Validation of the MLST scheme incorporated 422 additional public genomes: 401 downloaded from RefSeq and GenBank between February 2024 and 31 August 2025, and 21 genomes from this study (as detailed above) (Fig. S1b).

#### Implementation of ANI for accurate identification of *P. stuartii*

The MLST platform for *P. stuartii* (http://mlstps.insp.mx) incorporates the ANI tool to ensure the correct identification of *P. stuartii* from other species of *Providencia* genus. This tool enables users to verify the correct taxonomic assignment of candidate genomes prior to ST designation. The reference genomes for each bacterial species analyzed using ANI were *P. stuartii* BML2537 (NCBI RefSeq assembly number GCF_010320365.1), *P. rettgeri* FDAARGOS_1450 (NCBI RefSeq assembly number GCF_019048105.1), *P. alcalifaciens* DSM_30120 (GCF_000173415.1) and *P. hangzhouensis* PR-310 (GCF_029193595.2).

#### Molecular epidemiology of *P. stuartii* and metadata analysis

The molecular epidemiology of *P. stuartii* was determined using a total of 518 genomes (Fig. S1; Data set). The resistome was determined *in silico* using ABRicate software (24), which contains the Resfinder database (25). Moreover, plasmid replicon typing was determined using PlasmidFinder (26) and Mobile Element Finder (MEF) (27).

For the phylogenetic analysis of the seven concatenated MLST genes, IQ-TREE was used starting with the model finder to determine the substitution model that best fitted the data; therefore, an ML phylogeny was performed under the TIM3 +F + I + G4 substitution model.

#### goeBURST analysis

The goeBURST-1.2.1 program was used to analyze STs of *P. stuartii* isolates and to assign isolates to a clonal complex (CC). A CC is defined as groups of STs that have recently diversified into single-locus variants (SLVs) from a common founder (28). A CC was built of at least three STs related to a single locus variant.

#### Comparison of MLST schemes for *P. stuartii*

During the development of the *P. stuartii* MLST system described in this article, we noted that Arcari et.al (29) developed simultaneously a *P. stuartii* MLST system. To compare both schemes, we extract each of the gene fragments described in the Arcari system (*arnE*, *ftsH*, *gerA*, *rseA*, *tolR*, *yciA,* and *znuA*), and the STs were assigned among the 518 *P*. *stuartii* genomes. We designated our strain 15300 as ST1 and followed the same chronological assignment approach described in our scheme. A basic heatmap of the allelic profiles from both MLSTs was generated to visualize differences in locus variation. We evaluated both allele profiles using Mantel’s test, allele diversity per locus, and Simpson’s index. In addition, we also constructed an ML phylogenetic tree based on the core genome from the 518 genomes of *P. stuartii* that are part of the MLST, under the GTR + I + G model and assigned ST metadata to each scheme.

## RESULTS

### *P. stuartii* regional epidemiological surveillance study, carbapenemase identification and plasmid profile

Between 2017 and 2024, 70 clinical isolates of *P. stuartii* were recovered from seven institutions in Mexico. Of these, 37.1% (26/70) produced carbapenemases with NDM being the only variant identified (Table S2). Isolates were obtained from tracheal/bronchial secretions (24.4%), blood cultures (15.7%), wound samples (15.7%), secretion (14.3%), urine cultures (12.9%), expectoration (2.8%), stool culture (2.8%), tissue (1.4%), and unknown sources (10%) (Table S3). PFGE analysis revealed four major clonal groups, which were carriers of NDM associated with the Hospital Civil de Guadalajara. Plasmid profiling showed that most isolates harbored a 160-kb plasmid encoding NDM (Fig. S2). Conjugation assays confirmed the transfer plasmid-borne NDM to *E. coli* J53Azi^R^.

### Genome characteristics of *P. stuartii* clinical isolates

The 22 isolates confirmed as *P*. *stuartii* were sequenced (Fig. S1), which were obtained from bronchial/tracheal secretions (33.3%), blood cultures (33.3%), urine cultures (19.1%), wound swabs (9.5%), and secretion (4.8%) (Fig. S2). *P. stuartii* species classification was confirmed with ANI >99%. ResFinder analysis identified the NDM-1 in 14/22 genomes with 100% coverage and identity, consistent with PCR-based characterization. These strains carried plasmids of the IncA/C2 incompatibility group and mobile genetic elements including Tn7, IS5075, and ISCfr1. Genomic features of these isolates are detailed in Table S5.

### MLST scheme for *P. stuartii* and validation

Prior to the development of the MLST scheme, a curation of the 638 genomes of the *Providencia* genus was performed due to the identification of several inconsistencies in species assignment. Notably, genomes deposited under the name *P. thailandensis* clustered within *P. stuartii* clade with ANI >99%, confirming that both names correspond to the same species (Fig. S3). In contrast, another clade with genomes labeled as *P. stuartii* displayed ANI values well below the accepted species delineation threshold (83%–85%). Furthermore, pangenome analysis showed that although these groups shared most core genes, their accessory genomes were clearly distinct, supporting their classification as separate species (Fig. S4).

Based on ANI and SNP phylogeny, 95 genomes out of 638 were confirmed as *P. stuartii*, and together with the strain 15300 were used to identify complete single-copy genes for MLST. The scheme is composed of *rpoB* (RNA polymerase β-subunit), *tsf* (elongation factor Ts), *sucC* (succinyl-CoA synthetase β-subunit), *pflB* (formate acetyltransferase 1), *ftsA* (cell division protein FtsA), *dnaA* (chromosomal replication initiator protein DnaA), and *leuS* (leucyl-tRNA synthetase) (Table 1).

We determined 34 distinct STs among the 96 genomes used for the MLST development, whereas the MLST validation, using a dataset of 422 *P*. *stuartii* genomes, identified 70 additional STs. This resulted in a total of 104 STs among the 518 evaluated genomes.

### Molecular epidemiology of *P. stuartii*

The genomes of 518 *P*. *stuartii* isolates from various countries were primarily obtained from humans (88.2%), followed by insects (7%), environmental (1.2%), animals (0.6%), and unknown sources (3%) (Data set S1).

**Fig 1:**
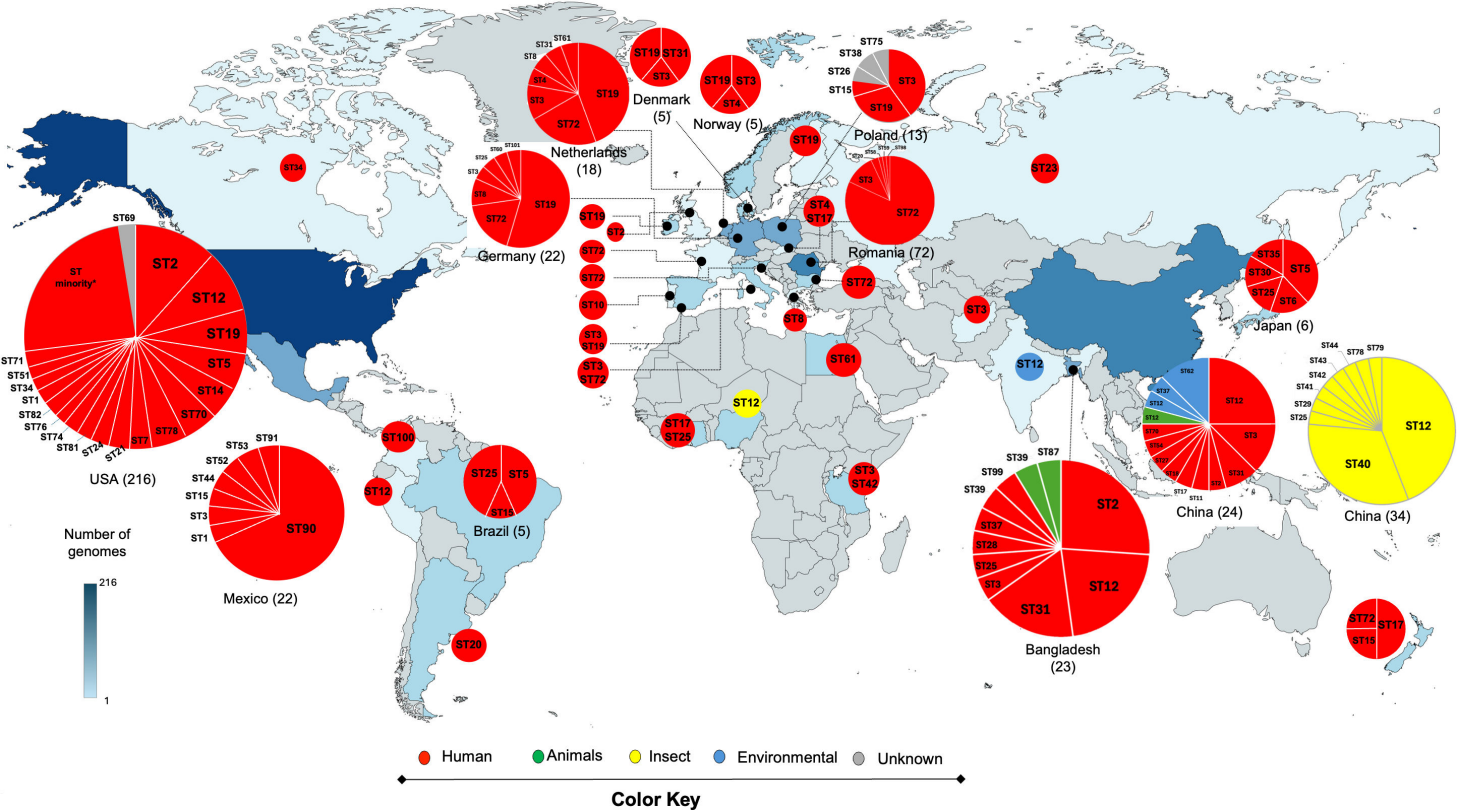
Molecular epidemiology of 518 *P*. *stuartii* isolates. The map illustrates the global distribution of each ST. The STs were distributed in pie charts according to the proportions of genomes from each of the corresponding countries. The origin of the isolates is indicated using color codes. ST72 exhibits the highest number of isolates, primarily in Romania, followed by Bulgaria, Netherlands, Germany, Slovenia, France, Italy, and New Zealand.

The global distribution of the STs is shown in Fig. 1 and Supplementary Data set S1. Some sequence types stand out, such as ST72, which is the most frequently identified (15.4% [*n* = 80/518]) and detected across several European countries and New Zealand. In addition, ST12 accounted for 10.4% (54/518) and was identified in countries from different continents and it was detected in all sample categories: humans, insects, environmental, animal, and one of unknown origin. ST3 had a worldwide distribution, being present in 14 different countries.

The United States, Romania, and China contributed the largest number of genomes. However, China exhibited the greatest diversity in isolation sources, including humans, insects, and wastewater. In contrast, genomes from the United States and Romania originated exclusively from human clinical samples. With respect to Mexico, ST1 derived from clinical samples, was also detected in the United States, whereas ST90 was the most frequent ST among Mexican human isolates.

### Molecular epidemiology of ESBL- and Carbapenemase-producing *P. stuartii*

**Fig 2:**
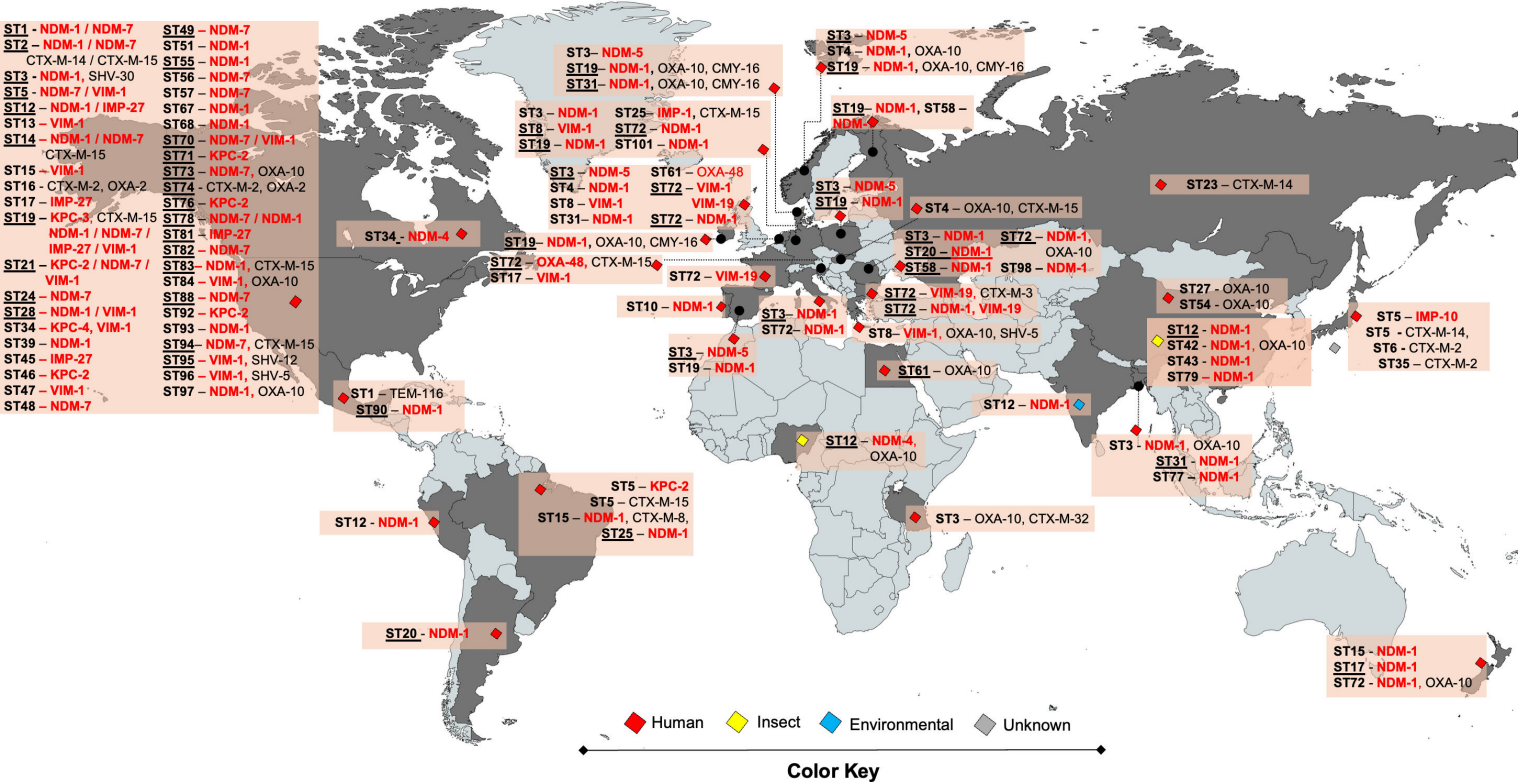
Molecular epidemiology of *P. stuartii* isolates producing ESBLs and carbapenemases distributed across 104 STs. The map illustrates the global distribution of 518 *P*. *stuartii*. Underlined and highlighted STs correspond to those with two or more isolates. The origin of the isolates is indicated using color codes. The USA, China, Brazil, Romania, Germany, Netherlands, and Bangladesh exhibited greater diversity of STs with various ESBLs and carbapenemases.

Of the 518 *P*. *stuartii* genomes analyzed, 66.4% contained at least one carbapenemase gene (Fig. 2; Fig. S5). The USA contributed the highest number of genomes (*n* = 216), assigned to different STs with substantial variability in carbapenemase gene content. In contrast, most human clinical samples from China lacked carbapenemase genes, except for two isolates encoding OXA-10, a β-lactamase with weak carbapenemase activity. Notably, ST72, one of the most common human-associated STs, showed a high prevalence of NDM-1, predominantly in Romania and other European countries. The ST19 (48 isolates) exhibited a notable diversity of carbapenemase genes and ESBLs, including members of the CTX-M family, and was distributed across nine countries. The ST90 (14 isolates), harboring NDM-1 and IncA/C2, was detected exclusively in Mexico.

A notable finding was the high proportion of isolates from flies that carried the NDM-1 or NDM-4 genes, primarily within ST12, with additional isolates belonging to ST42, ST43, and ST79. These genomes originated in China and Nigeria (Fig. 2), highlighting their potential epidemiological relevance.

In general, NDM-1 was the most prevalent carbapenemase gene (37.8%, 196/518), followed by NDM-7 (5.4%, 28/518). These NDM alleles were predominantly associated with ST72, ST12, ST19, ST3, and ST90 (Fig. S5). The ST19 (50 isolates) harbored NDM-1, NDM-7, KPC-3, IMP-27, and VIM-1. ST3 (36 isolates) comprised exclusively human clinical isolates and was associated with NDM-1 and NDM-5, while ST5 (13 isolates) carried primarily VIM-1 and NDM-7. Carbapenemases from the VIM, IMP, and KPC families were detected at lower frequencies compared with NDM.

Plasmid replicon typing identified different incompatibility groups, with IncA/C2 being predominant (60.4%, [*n* = 313/518]). This Inc group is known to have a broad host range among Enterobacterales and frequently carries multidrug-resistance modules, including NDM alleles. Consistent with this, IncA/C2 was strongly associated with isolates that carried carbapenem resistance genes in our dataset (Fig. S6).

**Fig 3:**
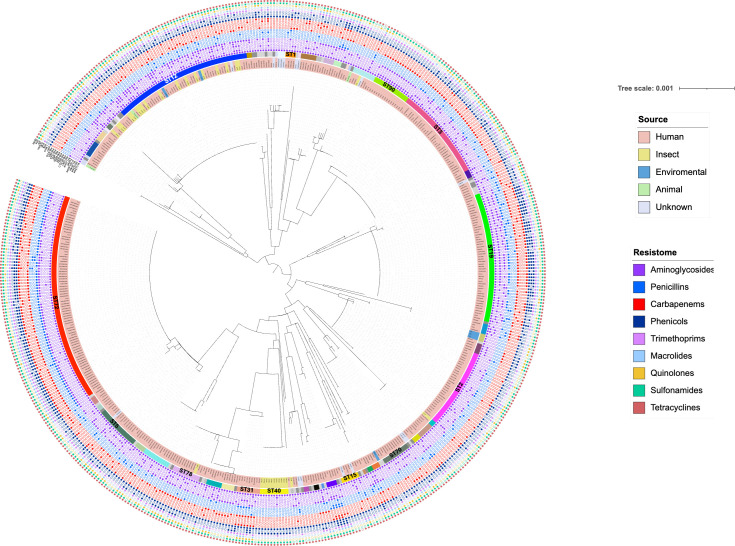
ML phylogenetic tree based on concatenated gene sequences (13,563 bp) from the MLST scheme for *P. stuartii*. Isolates are color-coded according to the sample origin. STs are highlighted and labeled with their respective names. The resistome present in each isolate is indicated by colored boxes, the presence of NDM is indicated by red boxes.

The phylogenetic analysis using the seven concatenated genes from the proposed MLST scheme revealed six main clusters, each corresponding to the predominant STs: ST72, ST12, ST19, ST3, ST2, and ST90 (Fig. 3). The metadata associated with these genomes indicated that *P. stuartii* commonly harbors resistance genes to multiple classes of antimicrobial, including fluoroquinolones, aminoglycosides, and β-lactams. Notably, genes associated with intrinsic resistance were present in more than 90% of the analyzed genomes, specifically *aac(2′)-Ia* (99.2%), *tet(B)* (92.3%), and *catA3* (94.4%). These highlight the resistome of *P. stuartii*, on which additional acquired genes, particularly NDM alleles, are frequently observed.

### Clonal complex and founder ST

The goeBurst analysis revealed a population structure characterized by the presence of several clonal complexes. The largest complexes were centered on ST72, ST1, ST12, and ST78, identified as founders, which showed multiple SLVs. CC1 dominated by ST72 corresponded to the most frequent ST in the total set of genomes analyzed. This complex included numerous closely related descendants (ST5, ST94, ST71, and ST103), which all were isolated from humans. Additionally, smaller clonal complexes were identified comprising those founded by ST25 and ST19 (Fig. S7).

### Comparison of MLST schemes

In 2024, Arcari et al. proposed a MLST scheme using seven gene fragments (*greA*, *ftsH*, *tolR*, *arnE*, *znuA*, *yciA*, and *rseA*) and a core genome MLST (cgMLST) based on 2,296 loci for the identification and typing of *P. stuartii*. However, we found some challenges in the implementation of their scheme, including the absence of a publicly accessible database for ST assignment; furthermore, it did not include the core genes used in their cgMLST. Their analysis was developed using 71 *P*. *stuartii* genomes.

To compare both systems, we extracted the gene fragments proposed by Arcari et al. and assigned STs to our collection of 518 *P*. *stuartii* genomes. During this process, we identified “N” insertions, which complicate allele identification, in the provided *greA* and *znuA* sequences. Since MLST is based on allelic variation, and “N” insertions represent four distinct variations. Therefore, to ensure consistency, we extracted the gene fragments using our reference genome (strain 15300) such as ST1.

**Fig 4:**
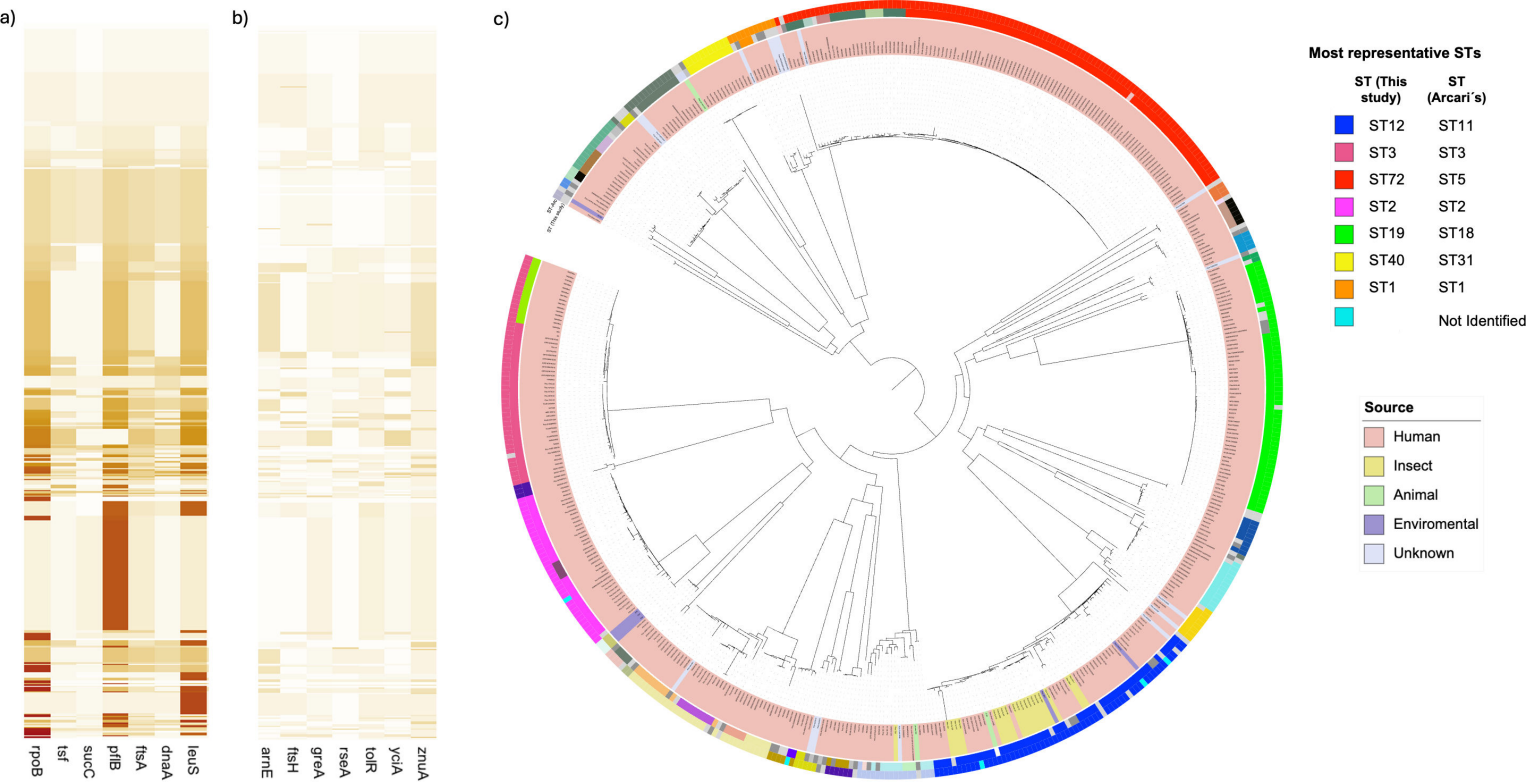
Heatmap and phylogenetic analysis showing allelic profile variation in *P. stuartii.* (**a**) Allelic profile variation of 518 *P*. *stuartii* genomes using the MLST scheme of this study. (**b**) Allelic profile variation of 513 *P*. *stuartii* genomes using the scheme of Arcari et al. (**c**) Phylogenetic analysis based on the core genome and compared with the ST distribution assigned with the two MLST schemes of *P. stuartii*.

Our MLST system assigned 104 STs among the 518 genomes, while Arcari’s scheme identified 55 STs among 513 genomes. Five genomes were excluded due to truncated or missing loci. The heatmap of allelic profiles showed greater heterogeneity with our MLST scheme compared with Arcari’s. This finding was supported by Simpson’s diversity index, which approached 1, suggesting sufficient genetic variation in our genes (Table S6). Mantel’s test revealed that both MLST systems captured similar patterns of strains, but the concordance is low (r = 0.1792, *P* = 1 × 10⁻⁴). However, the phylogenetic analysis based on core genome alignment showed that Arcari’s scheme was unable to capture slight differences in allele sequence from closely related isolates (Fig. 4).

## DISCUSSION

*P. stuartii* has emerged in recent years as an opportunistic pathogen with increasing reports of infections worldwide (30–33). In contrast, reports in Mexico have been scarce. In this study, clinical isolates from different institutions in the country were integrated to strengthen regional epidemiological surveillance of *Providencia* spp. We identified a high proportion of clinical isolates (37.1%) carrying the NDM-type carbapenemase, with clonal dissemination and persistence in the Hospital Civil de Guadalajara as demonstrated by PFGE. The presence of the NDM gene agrees with several reports worldwide that identified it as the main carbapenemase in *P. stuartii* (30, 32, 34), followed by IMP (35) and VIM (31). The relevance of this species lies in its intrinsic resistance to colistin and tigecycline, together with the acquisition of NDM and other carbapenemases, which confers resistance to carbapenems, limiting therapeutic options for these infections (33, 35–37).

In this context, the present study not only performed the molecular characterization of Mexican clinical isolates but also incorporated comparative analyses of *P. stuartii* genomes deposited in public databases to determine the molecular epidemiology worldwide; for this purpose, we developed a robust MLST system for *P. stuartii*, with an online public database for this bacterial species (http://mlstps.insp.mx). In this repository, a preliminary ANI analysis is performed to ensure accurate species identification.

During the process of development and genomic curation, we noted substantial taxonomic inconsistencies in RefSeq. Several genomes labeled as *P. thailandesis* correspond to *P. stuartii* (ANI >99%), a finding consistent with the recent taxonomic reclassification by Dong et al. (4). Conversely, genomes deposited as *P. stuartii* but belonging to recently described *P. zhejiangensis* (taxon 7) exhibited ANI values of 83%–85%, confirming misclassification. Although an ANI cut-off point of 98.21% was previously proposed to define *P. stuartii* (38), our analyses revealed that genomes truly belonging to this species consistently exceeded 99%, providing an even more precise delimitation. This highlights the need for continuous taxonomic curation in public repositories, particularly for genera undergoing rapid expansion and reclassification.

The traditional MLST, based on fragments of constitutive genes, requires labor-intensive and costly laboratory procedures, including DNA extraction, PCR amplification, purification, and Sanger sequencing for each locus (12). As the cost of WGS continues to decline, the MLST system proposed in this study based on the assignment of STs using seven full-length gene that allows capturing greater allelic variation, which enhances the robustness of the analysis and enables greater standardization and global comparability. Furthermore, access to complete genomes enables the simultaneous recovery of additional genomic features, such as the pangenome, resistome, and virulome that extend well beyond conventional ST assignments. Notably, we did not adopt a cgMLST approach due to variability in the core genome defining a fixed set of loci beings challenging because the number of shared genes can fluctuate depending on the size of the pangenome, genomic divergence (39), and assembly quality, which would affect reproducibility and comparison between studies. Using this scheme, we identified 104 STs among 518 genomes, the most comprehensive *P. stuartii* MLST to date, now accessible through an online public database (http://mlstps.insp.mx).

Our global epidemiological analysis revealed STs of relevance. ST72 emerged as the dominant clone and founder of CC1, mostly from human clinical isolates in Europe and frequently associated with NDM-type carbapenemases. ST12, in contrast, showed the broadest ecological distribution, present in human, insects, animal, and environmental samples. This wide ecological distribution underscores its potential for dissemination across ecological niches, reflecting its clonal expansion. In addition, ST19 and ST3 were associated with different carbapenemases and ST90, restricted to Mexico, was associated with NDM-1.

Notably, a several proportion of *P. stuartii* genomes carried plasmids with the IncA/C2 incompatibility group, which represents 60.4% of plasmid types. This plasmid family is widely recognized for its ability to disseminate NDM enzymes across species of Enterobacterales, and for its broad host range (40). The IncA/C2 is strongly associated with carbapenemase-positive isolates reinforcing its epidemiological importance.

Although most genomes originated from human sources, a significant subset (7%) belonged to house flies from China and Nigeria, which were associated with ST12 and carried the NDM gene. It has previously been reported that *P. stuartii* and other *Providencia* species are commonly found in the gut microbiota of house flies (9). Regarding the Nigerian flies, the carbapenemase NDM-4 was identified; these resulted from a pilot surveillance study of antimicrobial-resistant bacteria in Nigerian hospitals (41). The authors found NDM in 8% of the sampled flies, and it was primarily carried by *Providencia* species, such as *P. hangzhouensis, P. huaxiensis, P. manganoxydans, P. rettgeri,* and *P. stuartii*, along with *Enterobacter* spp., *E. coli*, and *K. pneumoniae*, among others. Furthermore, a national surveillance study conducted by Zhou et al. in China identified high rates of carbapenem-resistant Enterobacterales, of which *Providencia* spp. predominated with 90.6% attributed to the dissemination of NDM-1 (42). The detection of carbapenemase-producing *P. stuartii* in insects in China and Nigeria warrants further attention and highlights its relevance from a One Health perspective. This stems from the role of insects om the role of insects as a reservoirs and vehicles for the dissemination of resistance genes among various Enterobacterales and different ecological niches due to its frequent contact with wastewater, hospital environments, and decomposing organic matter. This threat is heightened in low- and middle-income countries, particularly in tropical climates, and may reflect the level of environmental contamination by carbapenem-resistant bacteria (41–43).

The resistome showed that nearly all genomes carried intrinsic resistance determinants, such as *aac(2')-Ia*, *tet(B*), and *catA3* (44, 45) and multiple acquired resistance genes, including ESBL and carbapenemase families (NDM, IMP, KPC, and VIM). The high diversity and prevalence of these genes emphasize the substantial genomic plasticity and acquisition potential of *P. stuartii*. Selective pressure in hospitals including prolonged use of tigecycline and colistin can that its emergence (46), especially considering the global expansion of NDM-1 and the presence of variants with greater carbapenem-hydrolyzing activity (47), such as NDM-7.

Although multiple MLST systems have been described for other pathogens, such as *A. baumannii* (48, 49) and *E. coli* (50–52), several authors adopt one system or include information from both MLST systems (53, 54). In the case of *P. stuartii*, we assume the same could occur. The *P. stuartii* MLST described in the present work provides greater robustness and enables comprehensive genomic insights beyond simple ST classification. Likewise, a public database update allows the determination of the bacterial species through ANI and the assignation of new sequence types.

### Conclusions

The development of MLST for *P. stuartii* enabled a comprehensive assessment of the global genetic diversity and population structure of this emerging multidrug-resistant pathogen. The scheme identified different STs distributed worldwide and epidemiologically relevant lineages grouped into clonal complexes. The ST72 and ST19 emerged as high-risk clones due to their strong association with carbapenemase genes, particularly NDM. ST12 is a globally distributed lineage detected across multiple ecological niches, including humans, environmental, and especially insects, where it frequently carried NDM alleles. Additional clinically relevant clones include ST90 prevalent in Mexico, and ST3 both demonstrated a broad international distribution. The widespread presence of IncA/C2 plasmid-type replicons among *P. stuartii* genomes highlights the importance of this incompatibility group as a reservoir of plasmids carrying NDM and other resistance determinants. Furthermore, the taxonomic classification in public databases underscores the need for genome-based approaches to ensure accurate species identification within *Providencia*. Overall, this study provides a tool for a more robust analysis and offers a general overview of the molecular epidemiology worldwide, emphasizing the importance of continuous genomic surveillance, particularly due to the acquisition of carbapenemase genes in the *P. stuartii*.

## Data Availability

The accession number of the genomes from this study was deposited under the BioProject “PRJNA1272119”.

## SUPPLEMENTAL MATERIAL

Figure S1 spectrum.02032-25-s0001.tiff Description of the epidemiological surveillance project.

Figure S2 spectrum.02032-25-s0002.tiff Dendrogram of clinical isolates of *Providencia stuartii*.

Figure S3 spectrum.02032-25-s0003.tiff Maximum likelihood phylogenetic tree.

Figure S4 spectrum.02032-25-s0004.tiff Distribution of pangenome.

Figure S5 spectrum.02032-25-s0005.tiff Circos plot linking the different STs.

Figure S6 spectrum.02032-25-s0006.tiff Distribution of Inc group in the different type sequences.

Figure S7 spectrum.02032-25-s0007.tiff goeBURST analysis of *P. stuartii* isolates.

Supplemental data set spectrum.02032-25-s0008.xlsx Metadata of the genomes.

Supplemental tables spectrum.02032-25-s0009.docx Tables S1, S2, S4, to S6.

Table S3 spectrum.02032-25-s0010.xlsx Clinical characteristics of *P. stuartii* isolates.

ASM does not own the copyrights to Supplemental Material that may be linked to, or accessed through, an article. The authors have granted ASM a non-exclusive, world-wide license to publish the Supplemental Material files. Please contact the corresponding author directly for reuse.
