# Supplementary figures and images for: Molecular epidemiology and population structure of Providencia stuartii obtained from humans and other sources
Source: Microbiol Spectr. 2025 Dec 31;14(2):e02032-25. doi: 10.1128/spectrum.02032-25 (PMC12889124; doi:10.1128/spectrum.02032-25)

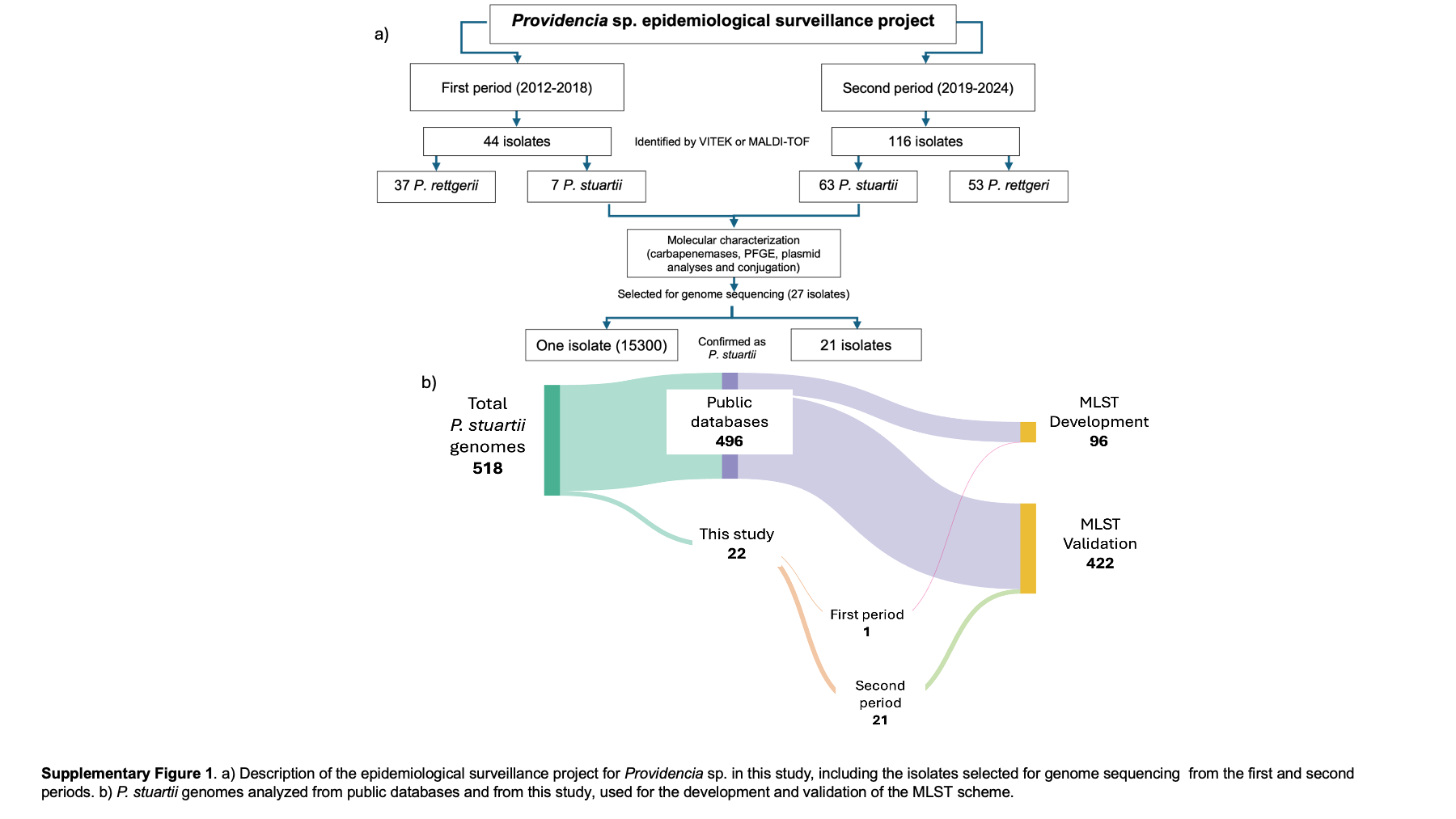

Supplement: Figure S1 — Description of the epidemiological surveillance project. [file spectrum.02032-25-s0001.tiff]

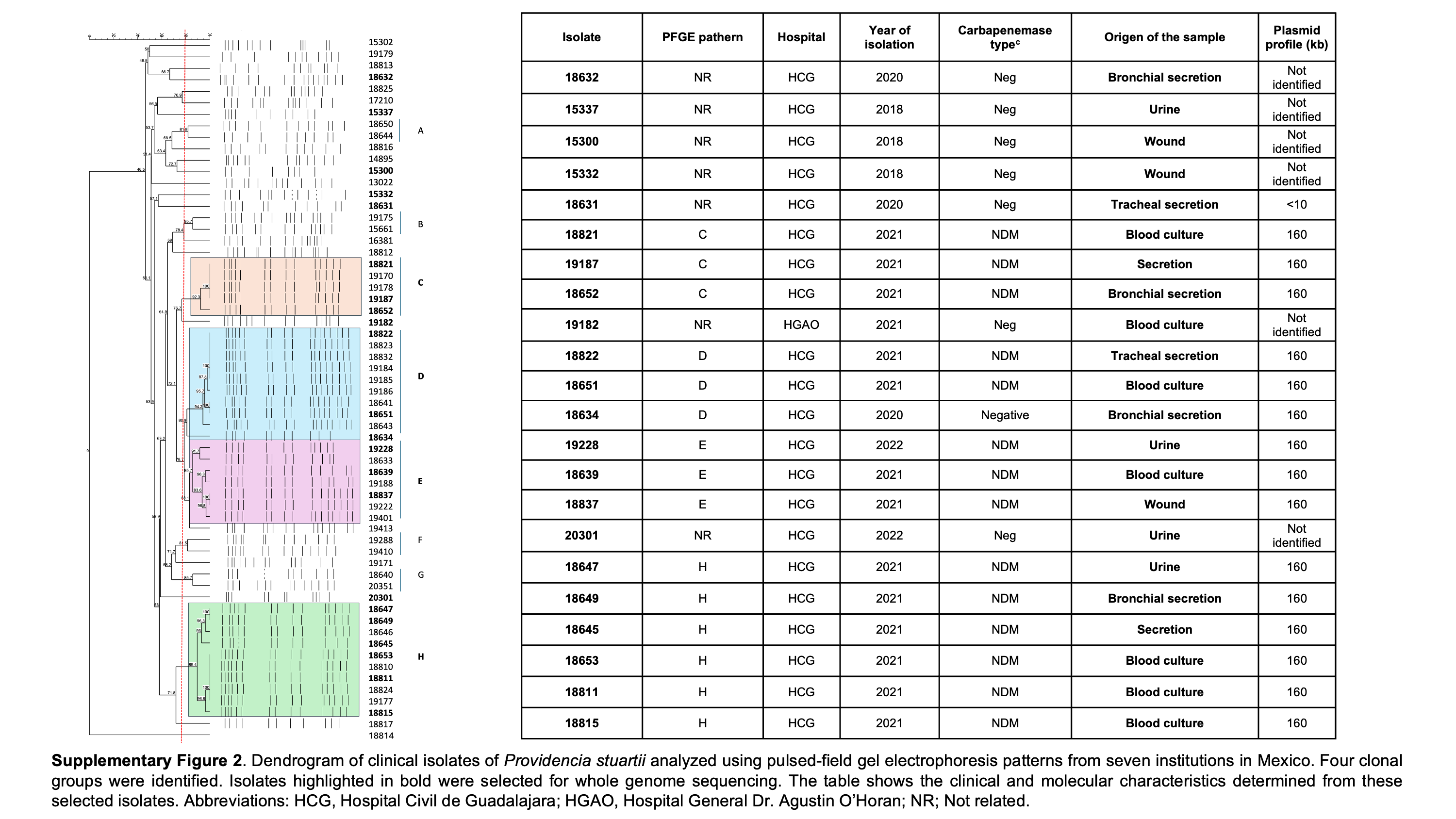

Supplement: Figure S2 — Dendrogram of clinical isolates of Providencia stuartii. [file spectrum.02032-25-s0002.tiff]

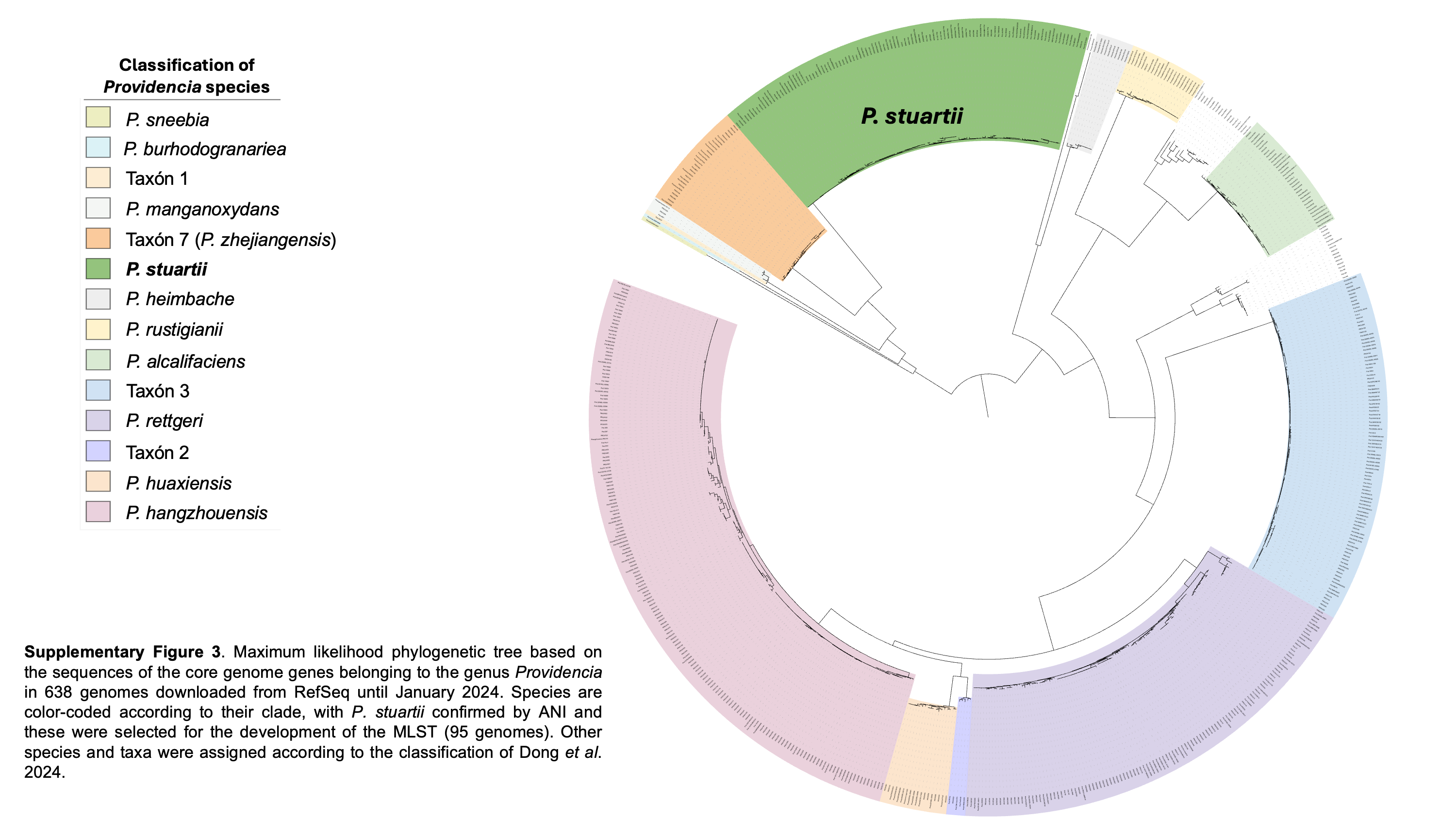

Supplement: Figure S3 — Maximum likelihood phylogenetic tree. [file spectrum.02032-25-s0003.tiff]

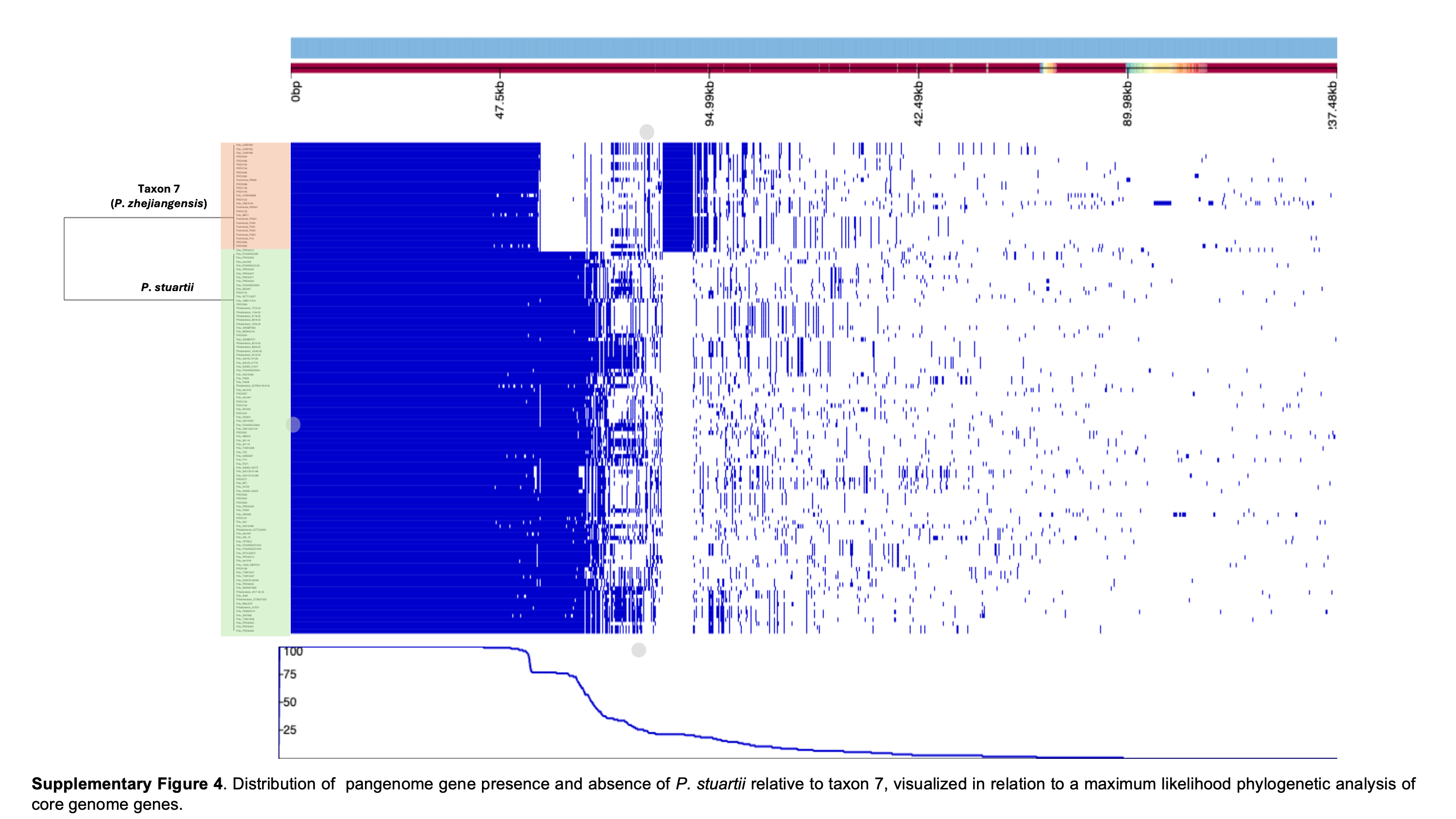

Supplement: Figure S4 — Distribution of pangenome. [file spectrum.02032-25-s0004.tiff]

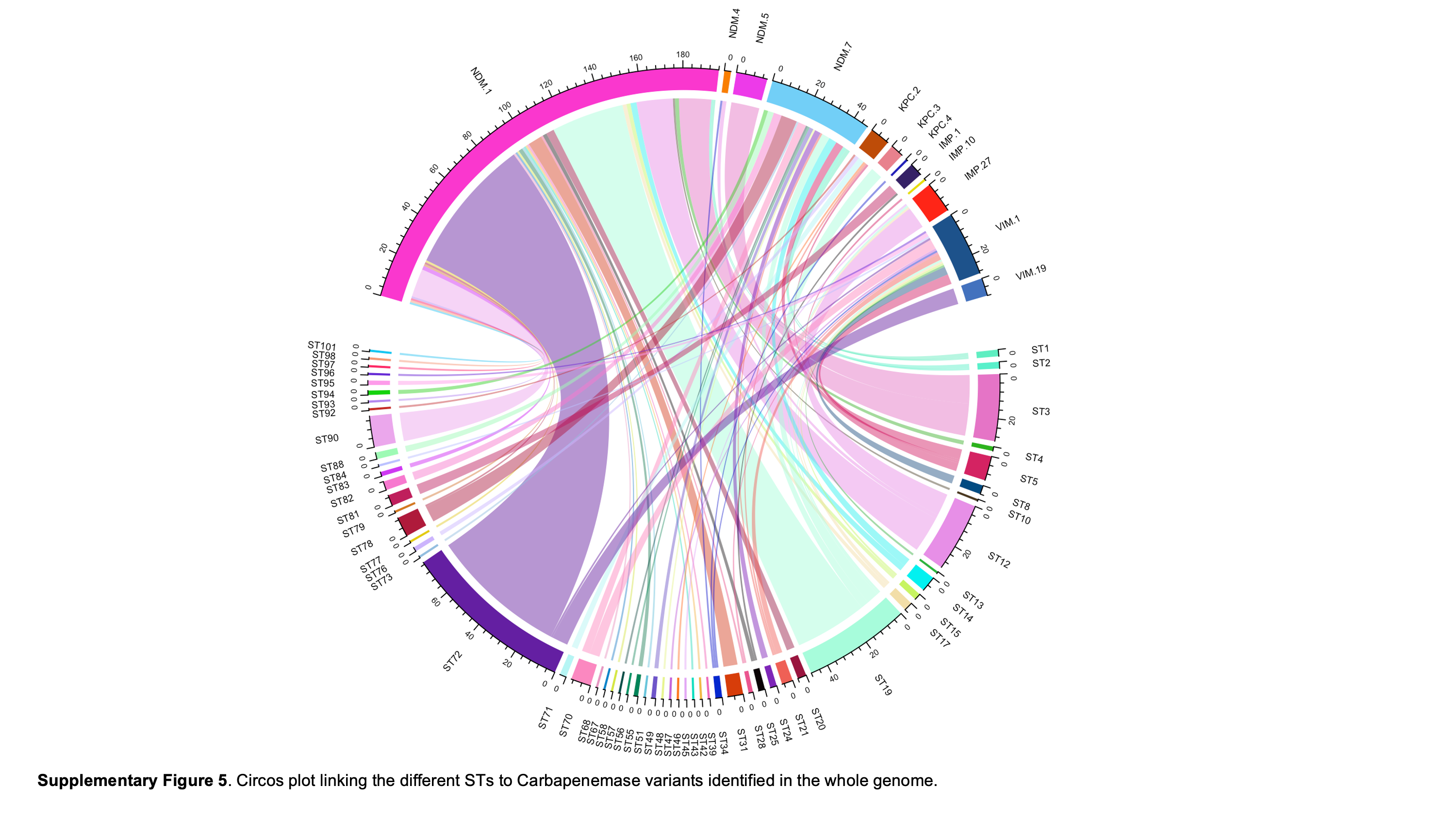

Supplement: Figure S5 — Circos plot linking the different STs. [file spectrum.02032-25-s0005.tiff]

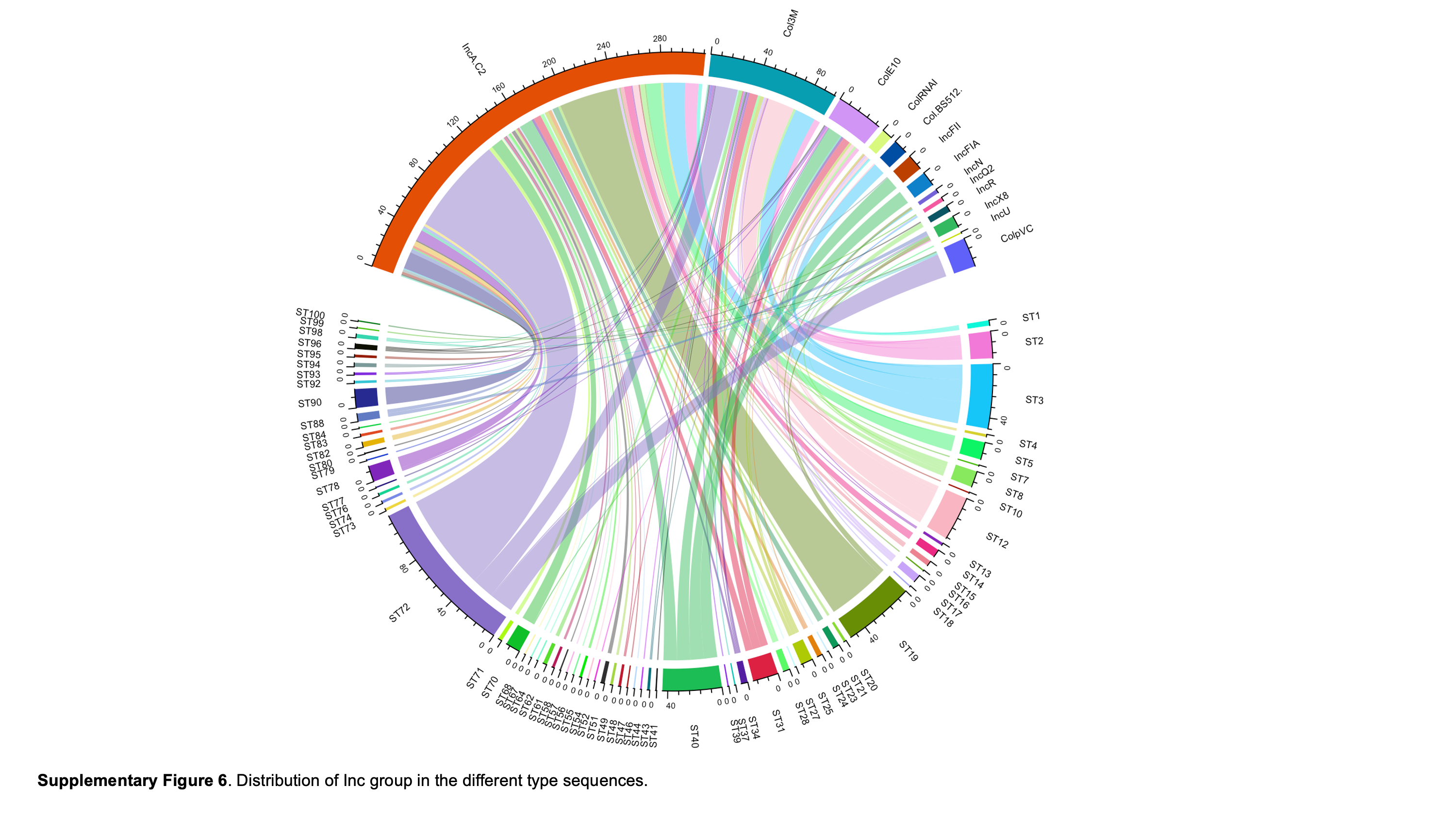

Supplement: Figure S6 — Distribution of Inc group in the different type sequences. [file spectrum.02032-25-s0006.tiff]

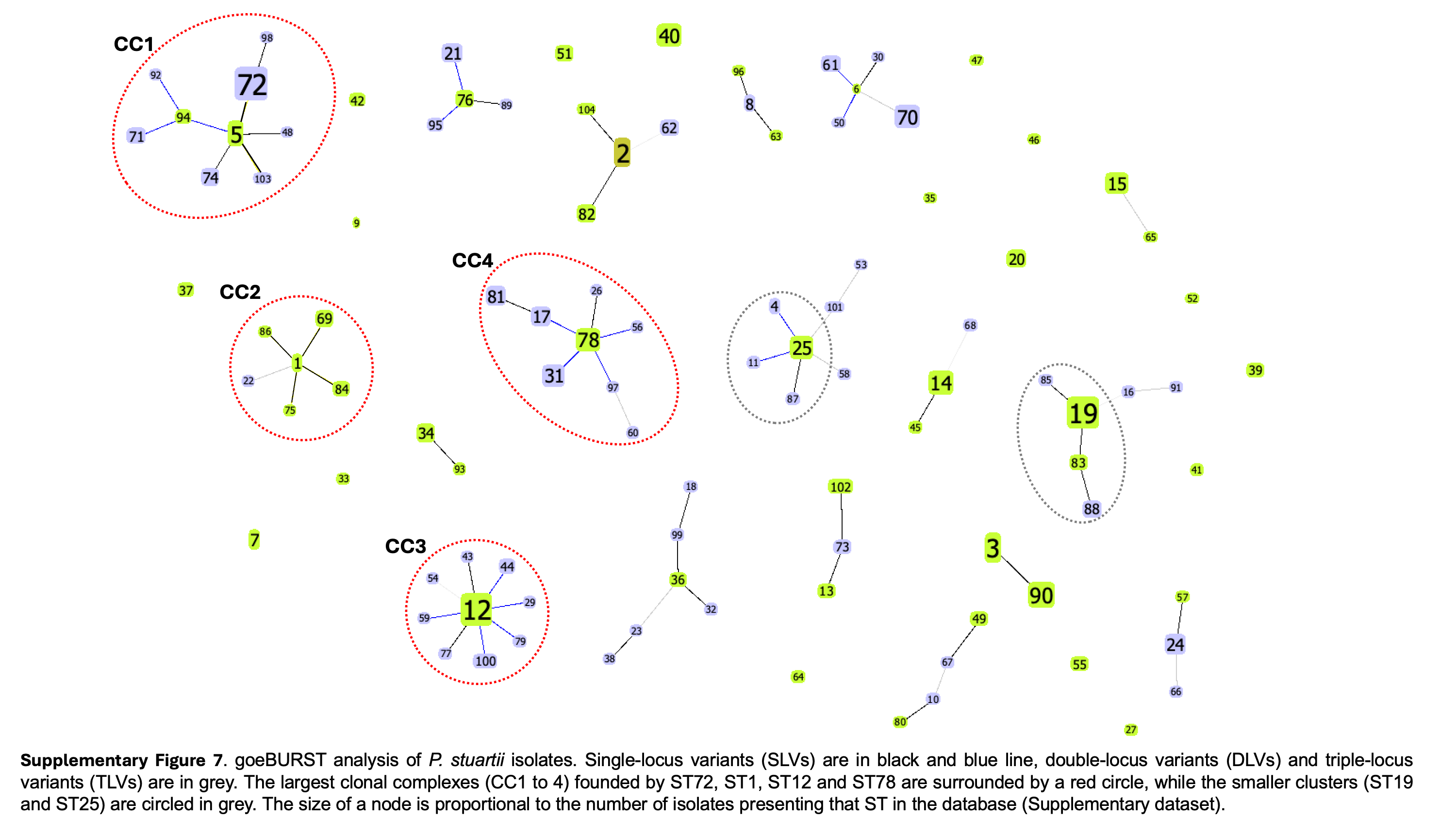

Supplement: Figure S7 — goeBURST analysis of P. stuartii isolates. [file spectrum.02032-25-s0007.tiff]
