# Supplementary material for: Molecular epidemiology and population structure of Providencia stuartii obtained from humans and other sources
Source: Microbiol Spectr. 2025 Dec 31;14(2):e02032-25. doi: 10.1128/spectrum.02032-25 (PMC12889124; doi:10.1128/spectrum.02032-25)
Supplement: Supplemental tables — Tables S1, S2, S4, to S6. [file spectrum.02032-25-s0009.docx]

**Supplementary Table 1**. Taxonomy of genus *Providencia* validated^a^

| **Specie** | **Taxonomy status** | **References** |
| --- | --- | --- |
| *P. stuartii* (1980) | Correct name | Ewing et al., 1962^1^, Skerman et al., 1980^2^ |
| *P. rettgeri* (1980) | Correct name | Brenner et al., 1978^3^, Skerman et al., 1980^2^ |
| *P. alcalifaciens* (1980) | Correct name | Skerman et al., 1980^2^ |
| *P. rustigianii* (1983) | Correct name | Hickman et al., 1983^4^ |
| *P. heimbache* (1986) | Correct name | Muller et al., 1986^5^ |
| *P. vermicola* (2006) | Correct name | Somvanshi et al., 1980^6^ |
| *P. burhodogranariea* (2009) | Correct name | Juneja et al., 2009^7^ |
| *P. sneebia* (2009) | Correct name | Juneja et al., 2009^7^ |
| *P. huaxiensis* (2019) | Correct name | Hu et al., 2019^8^ |
| *P. manganoxydans* (2022) | Correct name | Li et al., 2022^9^ |
| *P. huashanensis* (2024)^b^ | Correct name | Yang et al., 2024^10^ |
| *P. hangzhouensis* (2025) | Correct name | Dong et al., 2025^11^ |
| *P. zhijiangensis* | Correct name | Dong et al., 2025^12^ |
| *P. xianensis* (Taxon 3)^b^ | Correct name | Dong et al., 2024^13^ |
| *P. xihuensis* (Taxon 4) | Correct name | Dong et al., 2025^14^ |
| *P. zhejiangensis* (Taxon 7) | Correct name | Dong et al., 2025^14^ |
| *P. thailandesis*^c^ | Incorrect name | Kunthongpan et al., 2024^15^ |
| Taxon 1 | Names not defined | Dong et al., 2024^12d^ |
| Taxon 2 |  |  |
| Taxon 5 |  |  |
| Taxon 6 |  |  |

^a^ Adapted from LPSN (List of Prokaryotic names with Standing in Nomenclature).

^b^ *P. xianensis and P. huashanensis* are the same species. The official name has not yet been.

^c^ *P.* thailandesis is listed in LPSN as a valid species; however, it has now been confirmed to correspond to *P. stuartii.*

^d^ Following a taxonomic reclassification of the genus Providencia, the authors proposed seven new species, retaining the designations Taxon1-7 as provisional names.

REFERENCES:

1. Ewing, W. H. *Nomenclature and Taxonomy the Tribe Proteeae: Its Nomenclature and Taxonomy*. *BULLETIN OF BACTERIOLOGICAL* vol. 12 (1962).

2. Skerman VBD, M. V. S. PHA. Approved lists of bacterial names. *Int J Syst Bacteriol* **30**, 225–420 (1980).

3. Brenner,’, D. J. *et al.* *Deoxyribonucleic Acid Relatedness of Proteus and Providencia Species*. *INTERNATIONAL JOURNAL OF SYSTEMATIC BACTERIOLOGY* (1978).

4. Hickman-Brenner,’, F. W., Farmer Iii, J. J., Steigerwalt, A. G. & Brenner2, D. J. *Providencia Rustigianii: A New Species in the Family Enterobacteriaceae Formerly Known as Providencia Alcalifaciens Biogroup 3*. *JOURNAL OF CLINICAL MICROBIOLOGY* vol. 17 (1983).

5. Muller, H. E. *et al.* *Providencia Heimbachae, a New Species of Enterobacteriaceae Isolated from Animals*. *INTERNATIONAL JOURNAL OF SYSTEMATIC BACTERIOLOGY* vol. 36 (1986).

6. Somvanshi, V. S. *et al.* Providencia vermicola sp. nov., isolated from infective juveniles of the entomopathogenic nematode Steinernema thermophilum. *Int J Syst Evol Microbiol* **56**, 629–633 (2006).

7. Juneja, P. & Lazzaro, B. P. Providencia sneebia sp. nov. and Providencia burhodogranariea sp. nov., isolated from wild Drosophila melanogaster. *Int J Syst Evol Microbiol* **59**, 1108–1111 (2009).

8. Hu, Y., Feng, Y., Zhang, X. & Zong, Z. Providencia huaxiensis sp. Nov., recovered from a human rectal swab. *Int J Syst Evol Microbiol* **69**, 2638–2643 (2019).

9. Li, Z., Liao, F., Ding, Z., Chen, S. & Li, D. Providencia manganoxydans sp. nov., a Mn(II)-oxidizing bacterium isolated from heavy metal contaminated soils in Hunan Province, China. *Int J Syst Evol Microbiol* **72**, (2022).

10. Yang, W. *et al.* Identification of a novel Providencia species showing multi-drug-resistant in three patients with hospital-acquired infection. *Int J Antimicrob Agents* **64**, (2024).

11. Dong, X. *et al.* Whole-genome sequencing provides insights into a novel species: Providencia hangzhouensis associated with urinary tract infections . *Microbiol Spectr* **11**, (2023).

12. Dong, X., Jia, H., Yu, Y., Xiang, Y. & Zhang, Y. Genomic revisitation and reclassification of the genus Providencia . *mSphere* **9**, (2024).

13. Dong, X. *et al.* Novel Providencia xianensis sp. nov.: A multidrug-resistant species identified in clinical infections. *European Journal of Clinical Microbiology and Infectious Diseases* **43**, 1461–1467 (2024).

14. Dong, X., Xiang, Y., Shen, P., Xiao, Y. & Zhang, Y. Clinical emergence of Providencia zhejiangensis sp. nov. and Providencia xihuensis sp. nov.: Genomic insights into antimicrobial resistance and geographical distribution. *Int J Antimicrob Agents* **65**, (2025).

15. Khunthongpan, S., Sumpavapol, P., Tanasupawat, S., Benjakul, S. & H-Kittikun, A. *Providencia Thailandensis Sp. Nov., Isolated from Seafood Processing Wastewater*. *J. Gen. Appl. Microbiol* vol. 59 (2013).

16. Mulvey, M. R., Grant, J. M., Plewes, K., Roscoe, D. & Boyd, D. A. New Delhi metallo-β-lactamase in Klebsiella pneumoniae and Escherichia coli, Canada. *Emerg Infect Dis* **17**, 103–106 (2011).

17. Garza-Ramos, U. *et al.* Metallo-β-lactamase gene blaIMP-15 in a class 1 integron, In95, from Pseudomonas aeruginosa clinical isolates from a hospital in Mexico. *Antimicrob Agents Chemother* **52**, 2943–2946 (2008).

18. Hujer, K. M. *et al.* Analysis of antibiotic resistance genes in multidrug-resistant Acinetobacter sp. isolates from military and civilian patients treated at the Walter Reed Army Medical Center. *Antimicrob Agents Chemother* **50**, 4114–4123 (2006).

**Supplementary Table 2**. Clinical isolates of *P. stuartii* included in the study.

| **Center of origin (state)** | | ***Providencia* spp. surveillance study^b^** | **Number of isolates of *P. rettgeri*** | **Number of isolates of *P. stuartii*** | **Carbapenemase producing isolates^c^**  **n (%)** | |  | **Carbapenemase genes identified^d^ (%)** | |
| --- | --- | --- | --- | --- | --- | --- | --- | --- | --- |
|  |  |  |  |  | ***P. rettgeri*** | ***P. stuartii*** |  | ***P. rettgeri*** | ***P. stuartii*** |
| Hospitals belong to INVIFAR network^a^ | Hospital Civil de Guadalajara (Jalisco) | 113 | 51 | 62 | 2 (3.9%) | 26 (41.9%) |  | NDM (100) | NDM (100) |
|  | Instituto Nacional de Rehabilitación  (Ciudad de México) | 10 | 7 | 3 | 0 | 0 |  | 0 | 0 |
|  | Instituto Nacional de Cancerología  (Ciudad de México) | 5 | 4 | 1 | 0 | 0 |  | 0 | 0 |
|  | Hospital General de Zona N°1 IMSS Nueva Frontera Tapachula (Chiapas) | 1 | 0 | 1 | 0 | 0 |  | 0 | 0 |
|  | Hospital General del Estado “Dr. Ernesto Ramos Bours” (Sonora) | 3 | 2 | 1 | 0 | 0 |  | 0 | 0 |
|  | Hospital General “Dr. Agustin O’Horan” (Yucatán) | 3 | 2 | 1 | 0 | 0 |  | 0 | 0 |
|  | Hospital Universitario de Monterrey  (Nuevo León) | 21 | 21 | 0 | 19 (90.5%) | 0 |  | NDM (100) | 0 |
|  | Laboratorio Estatal de Michoacán  (Michoacán) | 1 | 0 | 1 | 0 | 0 |  | 0 | 0 |
|  | IMSS Monterrey (Nuevo León) | 1 | 1 | 0 | 1 (100%) | 0 |  | NDM (100) | 0 |
|  | Hospital Regional de Alta Especialidad Tapachula (Chiapas) | 1 | 1 | 0 | 0 | 0 |  | 0 | 0 |
|  | JM Research laboratorio (Morelos) | 1 | 1 | 0 | 0 | 0 |  | 0 | 0 |
|  | Total (%) | 160 | 90 (56.2) | 70 (43.8) | 22 (24.4) | 26 (37.1) |  | 100 | 100 |

^a^ All hospitals and institutes belong to INVIFAR network; the Hospital Civil de Guadalajara provided isolates prior to the participation of the rest of the hospitals or institutions in the network.

^b^ *Providencia* spp. surveillance included both *P. rettgeri* as *P. stuartii* identified by VITEK or MALDI-TOF.

^c^ Carbapenemase-producing isolates determined by CarbaNP (Review Materials and Methods).

^d^ Carbapenemase genes identified by PCR (Review Materials and Methods).

**Supplementary Table 4**. Oligonucleotides used in the study.

| **Gene** | **Primers (name: 5’-3’ sequence)** | **Size** | **References** |
| --- | --- | --- | --- |
| NDM-1 | NDM-F: GGT GCA TGC CCG GTG AAA TC  NDM-R: ATG CTG GCC TTG GGG AAC G | 660 pb | Mulvey *et al.,* 2011^16^ |
| IMP | IMP-F: GGA ATA GAG TGG CTT AAT TC  IMP-R: GCC AAG CTT CTA TAT TTG CG | 275 pb | Garza-Ramos *et al.*, 2008^17^ |
| VIM | VIM-F: GTG TTT GGT CGC ATA TCG C  VIM-R: CGC AGC ACC AGG ATA GAA G | 380 pb | Garza-Ramos *et al.*, 2008^17^ |
| OXA-24 | OXA-24F: ATG AAA AAA TTA TAC TTCC  OXA-24R: TTA AAT GAT TCC AAG ATTT C | 828 pb | Hujer *et al.,* 2006^18^ |
| OXA-58 | OXA-58F: AGT ATT GGG GCT TGT GCT  OXA-58R: AAC TTC CGT GCC TAT TTG | 453 pb | Hujer *et al.,* 2006^18^ |

References:

1. Mulvey, M. R., Grant, J. M., Plewes, K., Roscoe, D. & Boyd, D. A. New Delhi metallo-β-lactamase in Klebsiella pneumoniae and Escherichia coli, Canada. *Emerg Infect Dis* 17, 103–106 (2011).
2. Garza-Ramos, U. *et al.* Metallo-β-lactamase gene blaIMP-15 in a class 1 integron, In95, from Pseudomonas aeruginosa clinical isolates from a hospital in Mexico. *Antimicrob Agents Chemother* 52, 2943–2946 (2008).
3. Hujer, K. M. *et al.* Analysis of antibiotic resistance genes in multidrug-resistant Acinetobacter sp. isolates from military and civilian patients treated at the Walter Reed Army Medical Center. *Antimicrob Agents Chemother* 50, 4114–4123 (2006).

**Supplementary Table 5**. Genome characteristics of *P. stuartii* clinical isolates.

| **Isolate** | **Accession GenBank** | **CONTIGS (#)** | **N50** | **ANI** | **Carbapenemase** | **Inc group** | **Mobile Genetic Elements** |
| --- | --- | --- | --- | --- | --- | --- | --- |
| 15300 | GCF_050898035.1 | 250 | 48,530 | 99.51% | Negative | ColRNAI | ISEc46 (IS605) |
| 15332 | GCF_050895915.1 | 107 | 235,365 | 99.48% | Negative | Not Identified | ISVsa3 / ISEc75 (IS1595) / IS26 |
| 15337 | GCF_050898015.1 | 37 | 416,033 | 99.5% | Negative | Not Identified | ISEc46 (IS605) |
| 18631 | GCF_050896215.1 | 55 | 345,303 | 99.49% | Negative | ColE10 | ISEc75 (IS1595) |
| 18634 | GCF_050896155.1 | 63 | 387,935 | 99.99% | Negative | Not Identified | Tn7 |
| 18821 | GCF_050896095.1 | 63 | 375,538 | 99.99% | NDM-1 | IncA/C2 | Tn7 / IS5075 (IS110) / ISCfr1 (IS1182) |
| 19187 | GCF_050895675.1 | 68 | 290,864 | 99.99% | NDM-1 | IncA/C2 | Tn7 / IS5075 (IS110) / ISCfr1 (IS1182) |
| 18652 | GCF_050895735.1 | 70 | 375,539 | 99.99% | NDM-1 | IncA/C2 | Tn7 / IS5075 (IS110) / ISCfr1 (IS1182) |
| 18822 | GCF_050896075.1 | 75 | 375,642 | 99.99% | NDM-1 | IncA/C2 | Tn7 / IS5075 (IS110) / ISCfr1 (IS1182) |
| 18651 | GCF_050895775.1 | 75 | 375,538 | 99.99% | NDM-1 | IncA/C2 | Tn7 / IS5075 (IS110) / ISCfr1 (IS1182) |
| 18639 | GCF_050896195.1 | 73 | 375,641 | 99.99% | NDM-1 | IncA/C2 | Tn7 / IS5075 (IS110) / ISCfr1 (IS1182) |
| 18837 | GCF_050895705.1 | 75 | 375,642 | 99.46% | NDM-1 | IncA/C2 | Tn7 / IS5075 (IS110) / ISCfr1 (IS1182) |
| 18647 | GCF_050895835.1 | 73 | 375,538 | 99.99% | NDM-1 | IncA/C2 | Tn7 / IS5075 (IS110) / ISCfr1 (IS1182) |
| 18653 | GCF_050896175.1 | 79 | 375,642 | 99.99% | NDM-1 | IncA/C2 | Tn7 / IS5075 (IS110) / ISCfr1 (IS1182) |
| 18811 | GCF_050895475.1 | 71 | 375,539 | 100% | NDM-1 | IncA/C2 | Tn7 / IS5075 (IS110) / ISCfr1 (IS1182) |
| 18815 | GCF_050896115.1 | 71 | 375,539 | 99.46% | NDM-1 | IncA/C2 | Tn7 / IS5075 (IS110) / ISCfr1 (IS1182) |
| 19182 | GCF_050895555.1 | 47 | 386,541 | 99.97% | Negative | IncQ2 | Not Identified |
| 18649 | GCF_050896135.1 | 65 | 375,642 | 99.99% | NDM-1 | IncA/C2 | Tn7 / IS5075 (IS110) / ISCfr1 (IS1182) |
| 18645 | GCF_050895955.1 | 74 | 375,539 | 99.99% | NDM-1 | IncA/C2 | Tn7 / IS5075 (IS110) / ISCfr1 (IS1182) |
| 19228 | GCF_050896015.1 | 79 | 375,538 | 99.99% | NDM-1 | IncA/C2 | Tn7 / IS5075 (IS110) / ISCfr1 (IS1182) |
| 20301 | GCF_050895615.1 | 65 | 347,015 | 99.48% | Negative | Not Identified | IS30 / ISEc75 (IS1595) |
| 18632 | GCF_050895875.1 | 117 | 209,100 | 99.5% | Negative | Not Identified | IS3 / ISEc75 (IS1595) / IS26 |

**Supplementary Table 6**. Comparison of MLST schemes for *P. stuartii*.

| **MLST (This study)** | |  |  |
| --- | --- | --- | --- |
| **Gene** | **# alleles** | **Simpson’s Index of Diversity** | **CI (95%)** |
| *rpoB* | 58 | 0.911 | (0.900-0.923) |
| *leuS* | 48 | 0.913 | (0.900-0.926) |
| *pflB* | 49 | 0.925 | (0.915-0.934) |
| *dnaA* | 20 | 0.751 | (0.719-0.783) |
| *ftsA* | 19 | 0.873 | (0.860-0.885) |
| *sucC* | 13 | 0.720 | (0.693-0.746) |
| *tsf* | 16 | 0.672 | (0.630-0.714) |
| Number of strains used in this analysis: **518** | | | |

| **MLST Arcari’s** | |  |  |
| --- | --- | --- | --- |
| **Gene** | **# alleles** | **Simpson’s Index of Diversity** | **CI (95%)** |
| *znuA* | 16 | 0.711 | (0.679-0.743) |
| *yciA* | 13 | 0.681 | (0.653-0.709) |
| *tolR* | 10 | 0.746 | (0.729-0.764) |
| *rseA* | 12 | 0.527 | (0.482- 0.571) |
| *greA* | 10 | 0.727 | (0.704-0.751) |
| *ftsH* | 12 | 0.661 | (0.631-0.690) |
| *arnE* | 11 | 0.745 | (0.719-0.771) |
| Number of strains used in this analysis: **513** | | | |
